# A prospective evaluation of hidden bacterial involvement and antibiotic efficacy in nonbacterial CP/CPPS: addressing an underexplored therapeutic approach

**DOI:** 10.1007/s00345-025-05936-3

**Published:** 2025-09-15

**Authors:** Hiroyuki Kitano, Hiroki Ohge, Kayoko Tadera, Yuki Kohada, Tomoya Hatayama, Hiroyuki Shikuma, Ryo Tasaka, Kenshiro Takemoto, Shunsuke Miyamoto, Kohei Kobatake, Yohei Sekino, Hiroki Kitagawa, Seiya Kashiyama, Nobuyuki Hinata

**Affiliations:** 1 Department of Urology, Graduate School of Biomedical and Health Sciences, Hiroshima University, Hiroshima, 734-8551 Japan; 2 Department of Infectious Diseases, Hiroshima University Hospital, Hiroshima, 734-8551 Japan; 3 Department of Clinical Practice and Support, Hiroshima University Hospital, Hiroshima, 734-8551 Japan

**Keywords:** Antibacterial agents, Bacteriuria, Chronic pelvic pain, Prostatitis, Pyuria, Quality of life

## Abstract

**Purpose:**

To identify bacteria potentially involved in chronic prostatitis/chronic pelvic pain syndrome (CP/CPPS) classified as nonbacterial and evaluate the clinical effectiveness of antibiotic therapy against these pathogens.

**Methods:**

Patients were classified into two groups—CP/CPPS category IIIb and the bacterial prostatitis subgroup of CP/CPPS—based on the presence or absence of pyuria and bacteriuria in urine samples obtained before and after prostatic massage. Treatment efficacy was compared between the two groups. The bacterial prostatitis subgroup was further stratified according to whether uropathogens or non-uropathogens were detected, and treatment responses were analyzed accordingly.

**Results:**

A total of 28 patients were classified in the CP/CPPS category IIIb and 17 patients in the bacterial prostatitis subgroup. The bacterial prostatitis subgroup showed a significantly greater reduction in National Institutes of Health-Chronic Prostatitis Symptom Index total scores compared with the IIIb group, particularly in the pain domain, which also showed significant improvement over time. The quality-of-life scores also improved in this subgroup. Within the bacterial prostatitis subgroup, both uropathogen-positive and non-uropathogen-positive patients showed a reduction in pain scores, with significantly greater improvement observed in the non-uropathogen group.

**Conclusion:**

These findings suggest that some patients classified as CP/CPPS category IIIb under conventional diagnostic methods may, in fact, have bacterial involvement. Antibiotic therapy may be effective in such cases, including those with non-uropathogenic bacterial detection.

## Introduction

Chronic prostatitis/chronic pelvic pain syndrome (CP/CPPS), National Institutes of Health (NIH) category III, is a common genitourinary diagnosis in men, defined as the “presence of genitourinary pain in the absence of uropathogenic bacteria detected by standard microbiologic methodology” [1]. Pain associated with CPPS is primarily reported in the pelvis, perineum, and genital area and is often accompanied by lower urinary tract symptoms and sexual dysfunction. These symptoms can significantly reduce the patients’ quality of life (QOL) and contribute to depression and anxiety [2, 3].

Despite numerous studies [3–6] on the causes of CPPS, the exact etiology remains unclear. Some potential causes include infections, lower urinary tract dysfunction, autoimmune mechanisms, pelvic floor muscle spasms, or prostatitis [7]. CPPS often presents with symptoms similar to those of urinary tract infections and is frequently treated empirically with antibiotics. The presence of bacteria in the prostate has been demonstrated in earlier studies using transperineal prostate biopsy [8, 9]. However, it remains unclear whether the bacteria present in the prostate are involved in the pathogenesis of CPPS.

Based on the hypothesis that pathogens undetectable by conventional bacterial culture methods might be responsible for CPPS, studies have been conducted using different molecular techniques to identify hidden pathogens [4, 5]. Although the causative bacterial species have not been definitively identified, multiple bacterial species may be involved in the pathogenesis of CP/CPPS. This study aimed to clarify whether bacteria present in the prostate are involved in the pathophysiology of CPPS by identifying bacteria in the urine or expressed prostatic secretions of men and observing the efficacy of antibiotic treatment for CPPS-specific symptoms.

## Methods

### Study design and participants

This prospective study was approved by the Institutional Review Board of Hiroshima University (E2021-2509). The inclusion criteria were men with suspected CP/CPPS symptoms for at least 6 months and who had not used antibiotics for the past 3 months. The exclusion criteria were patients with NIH category I, II, or IV prostatitis syndrome, recent (4 weeks) antibiotic treatment, current use of prophylactic antibiotics, or the presence of indwelling transurethral or suprapubic catheter. The diagnosis of CP/CPPS was based on the NIH category III definition. Patients with ≥ 5 white blood cells per high-power field (HPF) in post-massage urine were classified as category IIIa, whereas those with 1–4 WBCs/HPF were classified as category IIIb [1]. Urinalysis of midstream urine was performed, but if pyuria was not detected, a prostate massage was performed for approximately 30 s. Post-massage urine and expressed prostatic secretion were then collected, with a total volume of at least 150 mL. These samples were used for urinalysis, general bacterial urine culture, and polymerase chain reaction (PCR) testing for *Mycoplasma hominis*, *Myc. genitalium*, *Ureaplasma parvum*, and *U. urealyticum*.

Patients in whom bacterial culture showed a bacterial count of ≥ 10³ CFU/mL or those who tested positive for any of the four pathogens by PCR were treated with appropriate antibiotics for approximately 6 weeks. Patients in whom no bacteria were detected underwent symptomatic treatment based on the UPOINTS (Urinary, Psychosocial, Organ Specific, Infectious, Neurological/systemic, and Tenderness of skeletal muscles) classification [10].

Before treatment and at 1, 2, and 3 months after treatment initiation, we obtained the patients’ NIH-Chronic Prostatitis Symptom Index (NIH-CPSI), International prostate symptom score (IPSS), and Overactive Bladder Symptom Score (OABSS) to evaluate treatment efficacy. We also obtained the patients’ responses to the following item from the G-8 geriatric screening tool: “In comparison with other people of the same age, how does the patient consider their health status?” [11].

### DNA extraction and PCR analysis

Urine samples were centrifuged at 13,000*g* for 10 min, and 400 µL of the sediment was used for DNA extraction. DNA was extracted using the automatic extraction system magLEAD^®^ with magLEAD^®^ Dx SV reagent (Precision System Science Co., Ltd., Chiba, Japan) in accordance with the manufacturer’s instructions. The final eluate (50 µL) was stored at − 80 °C until further use. The PCR test for *Myc. hominis* targeting 16 s rRNA gene was performed at the following conditions: 2 min at 95 °C; 30 cycles comprising 1 min at 95 °C, 1 min at 60 °C, and 1 min at 72 °C; and a final extension step of 5 min at 72 °C. The following PCR primers were used: RNAH1, 5′-CAATGGCTAATGCCGGATACGC-3′; RNAH2, 5′-GGTACCGTCAGTCTGCAAT-3′ [12]. The length of the *Myc. hominis* PCR product was 334 bp.

The PCR test for *Myc. genitalium* targeting 16 s rRNA gene was performed at the following conditions: 2 min at 95 °C; 35 cycles comprising 1 min at 95 °C, 50 s at 52 °C, and 50 s at 72 °C; and a final extension step of 5 min at 72°C. The following PCR primers were used: MgPa-1, 5′-AGTTGATGAAACCTTAACCCC7TGG-3′; MgPa-3, 5′-CCGTTGAGGGGTTTTCCATTTTTGC-3′ [13]. The length of the *Myc. genitalium* PCR product was 281 bp.

The PCR test for *U. parvum* was performed at the following conditions: 2 min at 95 °C; 30 cycles comprising 1 min at 95 °C, 1 min at 55 °C, and 1 min at 72 °C; and a final extension step of 5 min at 72 °C. The following PCR primers were used: UMS-57, 5′-CAAATCTTAGTGTTCATATTTTTTAC-3′; UMA-222, 5′-GTAAGTGCAGCATTAAAT TCAATG-3′ [3]. The length of the *U. parvum* PCR product was 327 bp.

The PCR test for *U. urealyticum* was performed at the same conditions as *U. parvum* using the following PCR primers: UMS-170, 5′-GTATTTGCAATCTTTATATGTTTTCG-3′; UMA-263, 5′- TTTGTTGTTGCGTTTTCTG-3′ [14]. The length of the *U. urealyticum* PCR product was 476 bp. The amplified fragments were analyzed on 2% (w/v) agarose gels containing ethidium bromide and visualized using an ultra-violet-transilluminator.

### Analysis of the 23 s rRNA gene

For the six samples confirmed positive for *Myc. genitalium* by PCR, the 23 S rRNA gene of *Myc. genitalium* was amplified and sequenced. The PCR for 23 S rRNA gene of *Myc. genitalium* was performed at the following conditions: 30 s at 98 °C; 30 cycles comprising 15 s at 98 °C, 15 s at 58 °C, and 15 s at 72 °C; and a final extension step of 5 min at 72 °C. The following PCR primers were used: 23 S-1930-F, 5′- TAACGGTCCTAAGGTAGCGA-3′; 23 S-2129-R, 5′-TCTCTACATGGTGGTGTTTTGA-3′ [15]. The length of the PCR product was 158 bp. Sanger sequencing in both directions was performed on DNA amplicons. To identify mutations/sequence variations, we compared DNA sequences with the reference sequence *Mycoplasma genitalium* G37 (GenBank accession no. NC_000908).

### Statistical analysis

Comparison between groups was performed using the Fisher’s exact and Mann–Whitney tests for categorical and continuous variables, respectively, using the JMP PRO18.1.0. (SAS Institute Inc., Cary, NC, USA). A two-way repeated measures analysis of variance (ANOVA) (type III sum of squares) was conducted to assess the effects of treatment over time between the two groups using GraphPad Prism version 10 software (Dotmatics Inc., San Diego, CA, USA). All tests were two-sided, and results were considered statistically significant at *p* < 0.05.

## Results

### Patient background characteristics

This study included a total of 51 Japanese men. Six patients who had bacteriuria in pre-massage urine samples and were diagnosed with category II chronic bacterial prostatitis were excluded from the study. Forty-five patients showed neither pyuria nor bacteriuria in pre-massage urine samples but reported symptoms of CP/CPPS. Among them, 28 patients also had no pyuria nor bacteriuria in post-massage urine and were diagnosed with category IIIb prostatitis (CP/CPPS category IIIb group). Their symptoms were classified according to the UPOINT system; treatment consisted of an α1-blocker or a phosphodiesterase type 5 inhibitor for urinary symptoms; Cernilton, Eviprostat, or acetaminophen for organ-specific symptoms; and pregabalin for tenderness of skeletal muscles. The remaining 17 patients showed no pyuria but did show bacteriuria in post-massage urine. Since these patients did not match into any existing NIH classification, they were categorized as the bacterial prostatitis subgroup of CP/CPPS. The clinical characteristics of all participants are summarized in Tables 1 and the clinical characteristics of patients with uropathogen or non-uropathogen-positive findings in the CP/CPPS bacterial prostatitis subgroup are summarized 2.

**Table 1:**
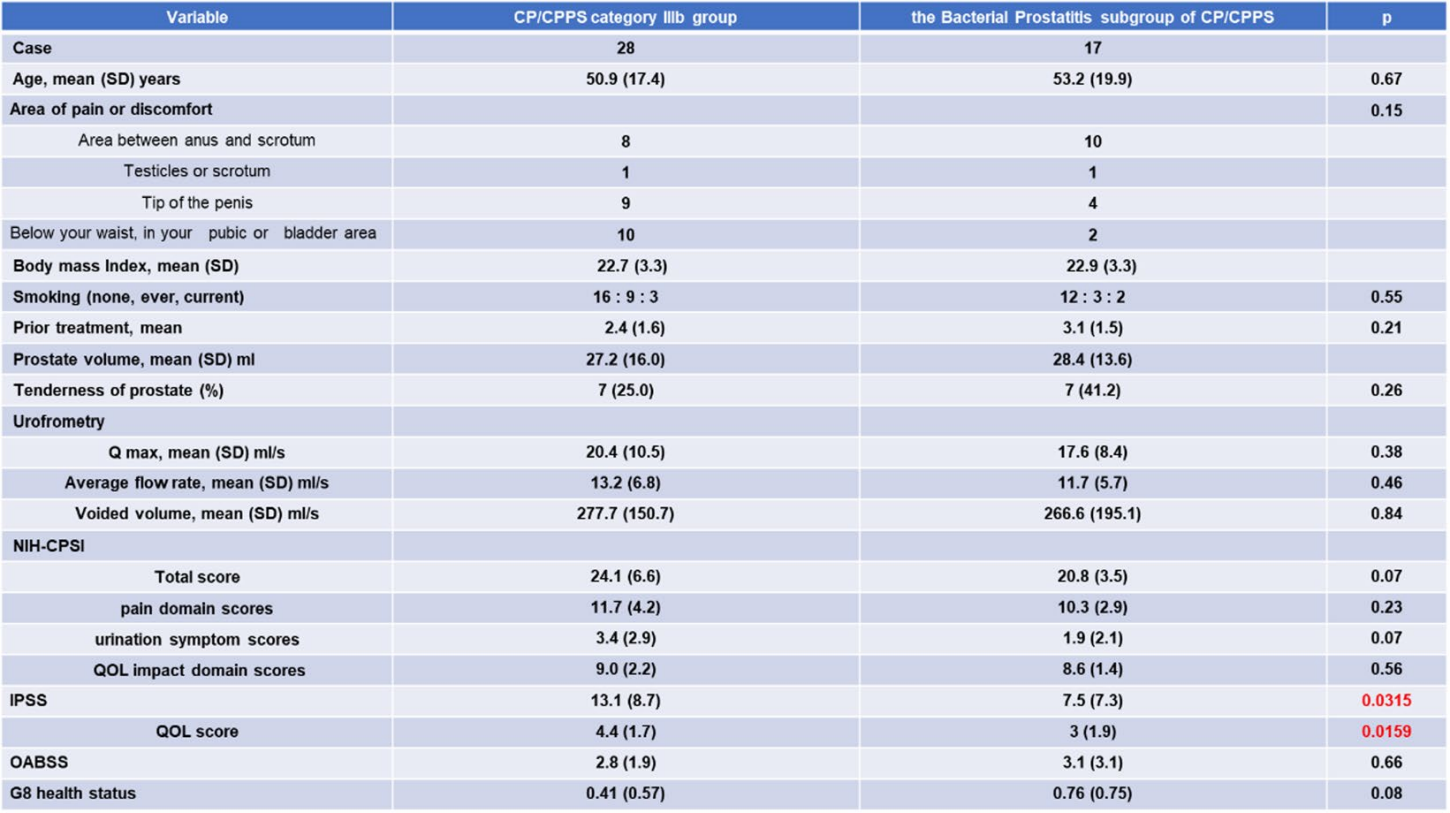
Baseline characteristics of patients with CP/CPPS category IIIb and the bacterial prostatitis subgroup of patients with CP/CPPS

**Table 2:**
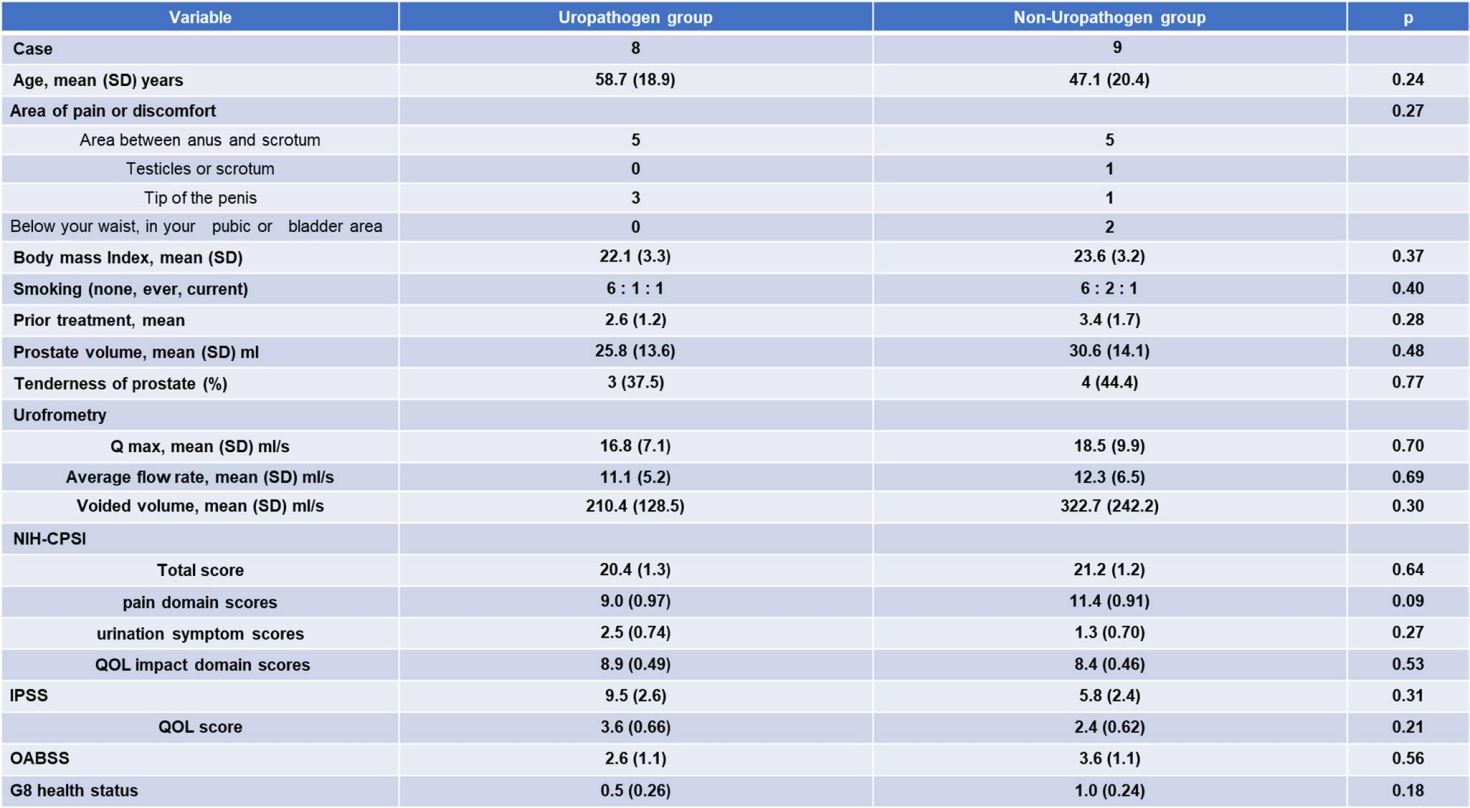
Baseline characteristics of patients with uropathogen or non-uropathogen detection in the CP/CPPS bacterial prostatitis subgroup

No significant differences were observed between the two groups in age, body mass index, number of previous treatments, prostate volume, prostate tenderness, uroflowmetry parameters (Qmax, Average flow rate, voided volume, residual volume), total NIH-CPSI score, and scores for pain, urinary symptoms, and QOL. However, the bacterial group showed significantly lower IPSS and QOL scores in IPSS. No significant differences were observed in OABSS and self-rated health status measured by Geriatric-8 screening tool.

### Bacteria

*Enterococcus faecalis* was the only bacterial species detected in five cases. *Mycoplasma genitalium*, *Citrobacter freundii*, and *Morganella morganii* were detected in three separate cases. These four bacteria were classified as uropathogens [16].

*Streptococcus mitis/oralis* was detected in two cases, whereas *Strep. agalactiae* and *Strep. hemolyticus* were detected in one case each. Furthermore, six additional cases showed the presence of *Strep. haemolyticus*, *Corynebacterium simulans*, *C. glucuronolyticum*, *Myc. hominis*, group B *Streptococcus*, and *Staphylococcus epidermidis*, respectively. These nine bacteria were classified as non-uropathogens [16].

### Antibiotics

Patients in whom *Enterococcus faecalis* was detected were treated with oral amoxicillin 500 mg every 8 h. The patient with *Myc. genitalium* was treated with oral azithromycin 1000 mg once weekly. The patient with *C. freundii* received oral amoxicillin/clavulanate 250/125 mg every 8 h, and the patient with *Mo. morganii* was treated with oral levofloxacin 500 mg once every 24 h. All treatments were continued for 6 weeks.

Patients in whom *Streptococcus* spp. were detected were treated with oral amoxicillin 500 mg every 8 h. Those with *Corynebacterium* spp. or *Myc. hominis* received oral levofloxacin 500 mg once every 24 h. All treatments were continued for approximately 6 weeks.

### Effectiveness

#### Comparison of the non-bacterial (CP/CPPS category IIIb) and bacterial prostatitis subgroup of CP/CPPS groups (Figs. 1)

A two-way repeated measures ANOVA (type III sum of squares) was applied to the changes in the total scores for NIH-CPSI and scores for other domains.

#### NIH-CPSI total scores

Total NIH-CPSI scores differed significantly between the groups (*P* < 0.0001). However, neither a significant effect of time (*P* = 0.3104) nor a group-by-time interaction (*P* = 0.5017) was found.

#### NIH-CPSI pain domain scores

Pain scores significantly differed between the groups (*P* = 0.0003); a significant effect of time was also observed (*P* = 0.0270). However, no significant group-by-time interaction was noted (*P* = 0.2221).

#### NIH-CPSI average of pain domain scores

A significant difference was observed among the levels of the row factor (*P* < 0.0001), while the column factor showed no significant effect (*P* = 0.1544). No significant interaction was found between the row and column factors (*P* = 0.3780).

#### Frequency of pain

Pain frequency significantly differed among the levels of the row factor (*P* = 0.0113), whereas no significant effect was observed for the column factor (*P* = 0.2114). The interaction between row and column factors was also not significant (*P* = 0.3760).

#### NIH-CPSI urinary symptom scores

No significant effects of group (*P* = 0.2987), time (*P* = 0.2448), or group-by-time interaction (*P* = 0.6600) were observed.

#### QOL impact domain scores

Significant main effects of group (*P* < 0.0001) and time (*P* = 0.0439) were observed. However, the group-by-time interaction was not significant (*P* = 0.1195).

#### IPSS scores

A significant group effect was observed (*P* = 0.0117), whereas the effects of time (*P* = 0.0976) and group-by-time interaction (*P* = 0.3218) were not significant.

#### IPSS QOL scores

No significant effects of group (*P* = 0.2911), time (*P* = 0.1441), or group-by-time interaction (*P* = 0.1339) were observed.

#### OABSS

No significant effects of group (*P* = 0.4185), time (*P* = 0.2978), or group-by-time interaction (*P* = 0.4593) were found.

#### G-8 health status domain

No significant effects of group (*P* = 0.6100), time (*P* = 0.3517), or group-by-time interaction (*P* = 0.7245) were observed.

### Comparison of NIH-CPSI total scores between uropathogens and non-uropathogens groups (Figs. 2)

A two-way repeated measures ANOVA (type III sum of squares) was performed to analyze differences in total scores for NIH-CPSI and scores for other domains over time between the two groups.

#### NIH-CPSI total scores

A significant difference was observed between the groups (*P* = 0.0024). However, no significant effect of time (*P* = 0.5547) or group-by-time interaction (*P* = 0.8304) was found.

#### NIH-CPSI pain domain scores

**Fig. 1:**
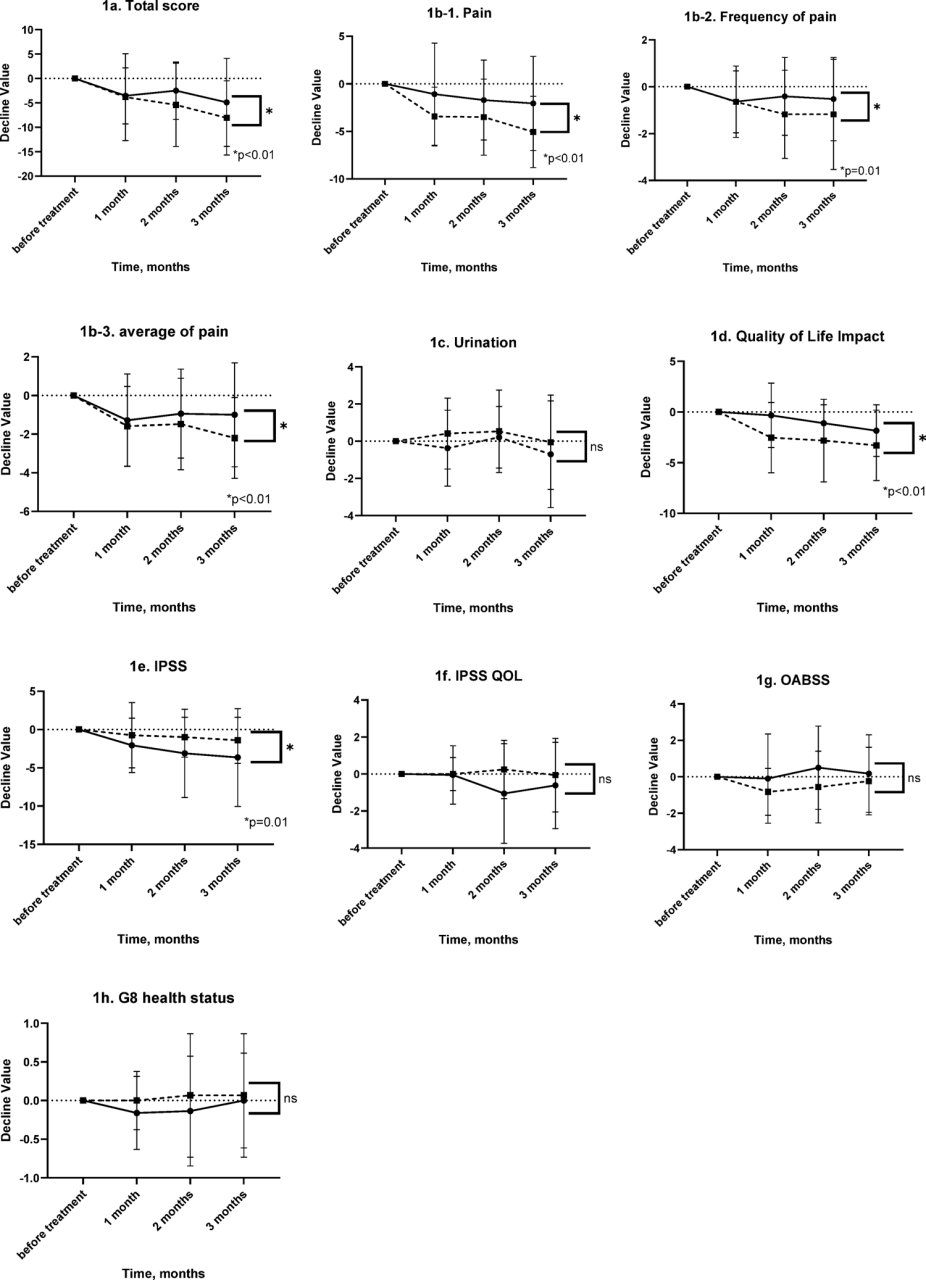
Time-course changes in symptom scores following treatment in patients with CP/CPPS category IIIb and in the bacterial prostatitis subgroup of CP/CPPS patients. Time-dependent changes in symptom scores over a 3-month period were compared between patients with CP/CPPS category IIIb and those in the bacterial prostatitis subgroup of CP/CPPS patients who received either symptomatic therapy or antibiotic treatment. **a** Total NIH-CPSI score, **b-1** NIH-CPSI pain domain score, **b-2** average pain score within the NIH-CPSI pain domain, **b-3** frequency of pain (NIH-CPSI), **c** NIH-CPSI urinary symptom score, **d** NIH-CPSI quality of life (QOL) impact domain score, **e** International Prostate Symptom Score (IPSS), **f** IPSS QOL score, **g** Overactive Bladder Symptom Score (OABSS), **h** G8 health status score. Symbols: ● Category IIIb group; ■ bacterial prostatitis subgroup

**Fig. 2:**
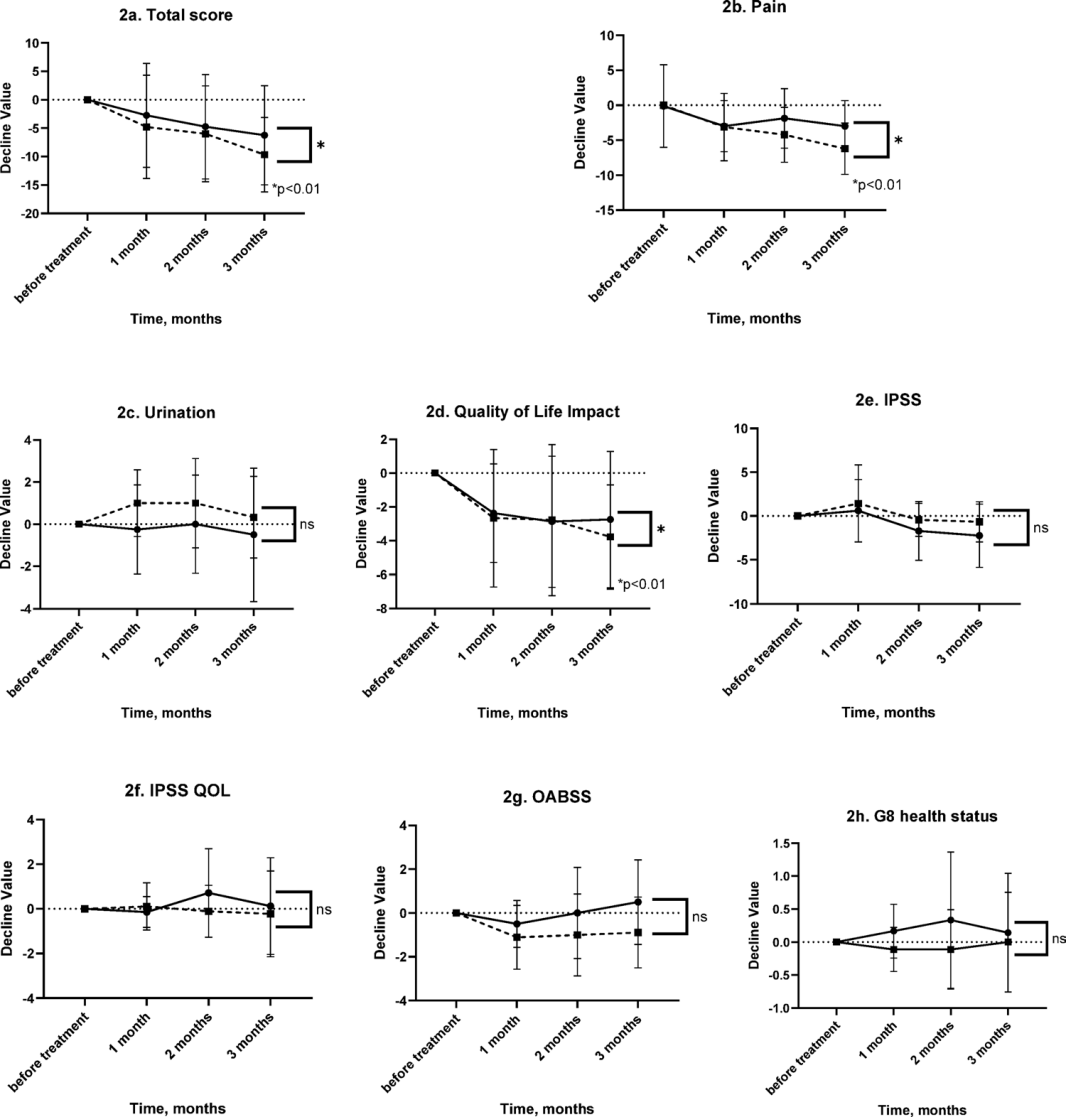
Time-course changes in symptom scores after antibiotic treatment in the bacterial prostatitis subgroup of CP/CPPS patients with uropathogen or non-uropathogen detection. Symptom changes over a 3-month period were compared between patients in the bacterial prostatitis subgroup of CP/CPPS patients who received antibiotic therapy, stratified by uropathogen or non-uropathogen detection. **a** Total NIH-CPSI score, **b** NIH-CPSI pain domain score, **c** NIH-CPSI urinary symptom score, **d** NIH-CPSI quality of life (QOL) impact domain score, **e** International Prostate Symptom Score (IPSS), **f** IPSS QOL score, **g** Overactive Bladder Symptom Score (OABSS), **h** G8 health status score. Symbols: ● Uropathogen group; ■ non-uropathogen group

Pain scores significantly differed between the groups (*P* = 0.0006). No significant effect of time (*P* = 0.3915) or group-by-time interaction (*P* = 0.2260) was observed (Figs. 1, 2).

#### Urination symptom scores

No significant effects of group (*P* = 0.5088), time (*P* = 0.3144), or group-by-time interaction (*P* = 0.5875) were found.

#### QOL impact domain scores

A significant difference was observed between the groups (*P* = 0.0030), while the effect of time (*P* = 0.8160) and the group-by-time interaction (*P* = 0.8806) were not significant.

#### IPSS

No significant effects of group (*P* = 0.0781), time (*P* = 0.2885), or group-by-time interaction (*P* = 0.7079) were observed.

#### IPSS QOL scores

No significant effects of group (*P* = 0.7151), time (*P* = 0.5710), or group-by-time interaction (*P* = 0.7192) were found.

#### OABSS

No significant effects of group (*P* = 0.2311), time (*P* = 0.1580), or group-by-time interaction (*P* = 0.3638) were observed.

### G-8 health status

No significant effects of group (*P* = 0.6220), time (*P* = 0.4324), or group-by-time interaction (*P* = 0.5807) were found.

### Adverse events

No adverse events, including fever or pain, occurred after prostatic massage. A 6-week course of antibiotic treatment was administered to the bacterial group, but no resistant bacteria or *Clostridioides difficile* infection were observed.

## Discussion

The prevalence of CPPS has been reported to be 8% in community-based surveys [17], with some estimates reaching as high as 11.5% among men under the age of 50 [18], indicating that it is a relatively common benign condition.

However, the pathophysiology of CPPS remains unclear. Although several studies have investigated the potential involvement of bacteria in CPPS [4, 9], our study is the first to use liquid culture media for bacterial detection and to compare outcomes between patients treated with antibiotics—after bacterial identification—and a control group. Among the bacteria detected in our study, the most frequently identified uropathogen was *E. faecalis*, found in 5 cases (62.5%). This finding is consistent with previous reports in which *E. faecalis* was detected in 30–80% of uropathogen-positive cases [4, 16], suggesting that it may be a predominant species in bacterial CPPS cases. As observed in the present study, bacterial species detected in CPPS differ from those typically observed in complicated urinary tract infections, such as gram-negative bacilli—*Escherichia coli* and *Klebsiella pneumoniae* [19]. Many of the detected organisms are so-called “non-uropathogens,” which are generally considered non-pathogenic in the urinary tract. Werneburg et al. [4] identified *Mycoplasma penetrans in one case*, whereas we detected *Myc. genitalium* in one case with no prior history of pyuria or bacteriuria. *Mycoplasma genitalium* was detected for the first time through PCR test on post-prostatic massage urine. Although reports of *Myc. genitalium* in patients with CPPS are limited, Krieger et al. performed PCR tests on prostate biopsies from 135 patients diagnosed with CPPS and detected *Myc. genitalium*, *Chlamydia trachomatis*, and *Trichomonas vaginalis* in 8 cases. These findings suggest that sexually transmitted pathogens may also be involved in the pathogenesis of CPPS [20].

We observed that urinary function tended to be better in the group with bacterial detection than in the one without bacterial detection. However, despite no significant difference in the total NIH-CPSI score, the pain score tended to be relatively higher in the group with bacterial detection. Similar to the findings by Werneburg et al. [4], we found no significant differences in any of the NIH-CPSI domains between patients with and without detection of uropathogens.

Although the presence of bacteria in the prostate has been demonstrated, CPPS often presents with symptoms similar to those of urinary tract infections and is frequently treated empirically with antibiotics despite limited evidence. Reports of antibiotic treatment specifically targeting causative pathogens are extremely limited [4, 7, 21].

In their study, Werneburg et al. reported that among nine individuals in whom bacteria were detected via NGS, only one (11%) did not show therapeutic response to antibiotic treatment [4]. They defined a positive treatment response as the disappearance of pain and lower urinary tract symptoms 6 weeks post-treatment. Another study evaluating antibiotic efficacy for chronic bacterial prostatitis reported temporary symptom improvement in 40 (83.3%) of 48 patients [22]. In our comparison between groups with and without bacterial detection in CPPS, significant pain improvement with antibiotic therapy was observed in the bacterial detection group. These findings suggest that antibiotics may be effective in patients with CPPS where bacteria are implicated. Intraprostatic bacteria may persist due to biofilm formation, allowing symptoms to continue even in the presence of few leukocytes in the urine; antibiotic treatment can be effective in such cases [22]. The duration of antibiotic therapy, typically reported as 4–6 weeks [4, 23], remains an area for future investigation.

The contribution of microbial infection to conditions such as CP/CPPS and interstitial cystitis/bladder pain syndrome has remained unclear [24]. However, recent findings using NGS, as well as highly sensitive bacterial culture techniques, have demonstrated the presence of a bladder microbiome. A decrease in the bladder microbiome diversity has been associated with interstitial cystitis/bladder pain syndrome [25].

In the present study, we employed liquid culture media, instead of blood agar plates, for bacterial cultivation, resulting in a positive detection rate of 37.8% (17/45). This detection rate is comparable to that reported using prostate biopsy [8] or NGS [4], suggesting that our method may also be useful for identifying bacteria in patients with CPPS. Nickel et al. used the Ibis T-5000 Universal Biosensor system—a highly sensitive hybrid system that combines PCR with mass spectrometry (MALDI-TOF MS), allowing for the simultaneous identification of multiple microbial species without the need for culture—to analyze the urinary microbiota in initial and midstream urine samples from women with CPPS [26]. Although they observed no difference in the number of detected bacterial species between the flare and non-flare groups, fungi—specifically *Candida* and *Saccharomyces* species—were significantly more prevalent in the flare group [27]. Nickel et al. also conducted a comparative analysis using the Ibis T-5000 Universal Biosensor system to assess the urinary microbiota in men with CP/CPPS and asymptomatic controls. They analyzed first-void, midstream, and post-prostatic massage urine samples. While no significant difference was observed in the mean number of detected microbial species per individual between the patients with CPPS and controls, a significant difference in microbial composition was identified at both the species and genus levels (*p* = 0.002 and *p* = 0.004, respectively). Notably, *Burkholderia cenocepacia* was significantly more frequently detected in the CPPS group [27].

This study is limited by the relatively short observation period. Nevertheless, the findings offer clinically valuable insights into the diagnosis and treatment of CPPS.

Although none of the patients showed symptoms suggestive of urethritis, such as purulent discharge, the potential presence of urethral-derived *Mycoplasma* spp. cannot be ruled out as 16S rRNA analysis was not conducted before prostate massage. While a certain therapeutic effect was observed with antibiotic treatment, post-treatment culture testing was not performed. Re-examination with both culture and 16S rRNA PCR testing may be necessary to confirm bacterial eradication.

*Mycoplasma hominis*, *U. parvum*, and *U. urealyticum* are commensal bacteria associated with colonization and infection of the urogenital tract [28]. In the present study, metagenomic analysis of post-massage urine or EPS might also have enabled a more comprehensive characterization of the prostatic microbiota.

## Conclusion

Our study demonstrated the presence of bacteria in cases of CPPS and showed that antibiotic administration can alleviate pain in affected patients. Although treatment for CPPS is generally symptomatic due to the heterogeneous clinical manifestations, our findings suggest that antibiotic therapy is effective when bacteria are detected and may represent a valuable treatment strategy. Future research should focus on determining the optimal duration of antibiotic treatment.

## Data Availability

No datasets were generated or analysed during the current study.
